# Safety of deep brain stimulation in pregnancy: A comprehensive review

**DOI:** 10.3389/fnhum.2022.997552

**Published:** 2022-09-29

**Authors:** Caroline King, T. Maxwell Parker, Kay Roussos-Ross, Adolfo Ramirez-Zamora, John C. Smulian, Michael S. Okun, Joshua K. Wong

**Affiliations:** ^1^Department of Obstetrics & Gynecology, University of Florida College of Medicine, Gainesville, FL, United States; ^2^Department of Neurology, Norman Fixel Institute for Neurological Diseases, University of Florida College of Medicine, Gainesville, FL, United States; ^3^Center for Research in Perinatal Outcomes, University of Florida College of Medicine, Gainesville, FL, United States

**Keywords:** pregnancy, DBS, clinical trials, safety, ethics, deep brain stimulation, neuromodulation

## Abstract

**Introduction:**

Deep brain stimulation (DBS) is increasingly used to treat the symptoms of various neurologic and psychiatric conditions. People can undergo the procedure during reproductive years but the safety of DBS in pregnancy remains relatively unknown given the paucity of published cases. We thus conducted a review of the literature to determine the state of current knowledge about DBS in pregnancy and to determine how eligibility criteria are approached in clinical trials with respect to pregnancy and the potential for pregnancy.

**Methods:**

A literature review was conducted in EMBASE to identify articles involving DBS and pregnancy. Two reviewers independently analyzed the articles to confirm inclusion. Data extracted for analysis included conditions treated, complications at all stages of pregnancy, neonatal/pediatric outcomes, and DBS target. A second search was then conducted using www.clinicaltrials.gov. The same two reviewers then assessed whether each trial excluded pregnant individuals, lactating individuals, or persons of childbearing age planning to conceive. Also assessed was whether contraception had to be deemed adequate prior to enrollment.

**Results:**

The literature search returned 681 articles. Following independent analysis and agreement of two reviewers, 8 pregnancy related DBS articles were included for analysis. These articles described 27 subjects, 29 pregnancies (2 with subsequent pregnancies), and 31 infants (2 twin pregnancies). There was 1 preterm birth at 35 weeks, and 3 patients who experienced discomfort from the DBS battery (i.e., impulse generator) placement site. All 27 patients had a DBS device implanted before they became pregnant, which remained in use throughout their pregnancy. There was exclusion of pregnant individuals from 68% of 135 interventional trials involving DBS. Approximately 44% of these trials excluded persons of childbearing age not on “adequate contraception” or wishing to conceive in the coming years. Finally, 22% excluded breastfeeding persons.

**Conclusion:**

The data from 29 pregnancies receiving DBS treatment during pregnancy was not associated with unexpected pregnancy or post-partum complication patterns. Many clinical trials have excluded pregnant individuals. Documentation of outcomes in larger numbers of pregnancies will help clarify the safety profile and will help guide study designs that will safely include pregnant patients.

## Introduction

Deep brain stimulation (DBS) has been applied selectively for treatment of Parkinson’s disease (PD), essential tremor (ET), dystonia, and many other neuropsychiatric disorders and symptoms (Denison and Morrell, 2022). The steadily improving safety profile has led to expansion into younger and healthier populations during reproductive ages, which raises important issues regarding DBS and pregnancy. Individuals with DBS may be interested in becoming pregnant or may unknowingly discover that they are pregnant after a DBS device has been implanted. Others may also be interested in the safety profile during lactation and whether or not they should consider enrollment in a clinical trial if pregnant or considering a future pregnancy.

There has been an increase in clinical trials utilizing DBS across 28 conditions, many of which are in the psychiatric or cognitive disease domains (Harmsen et al., 2020). Many focus on younger patients and thus pregnancy-related issues become more pertinent (Schrag and Schott, 2006; Louis and Dogu, 2008). With mean maternal age at first birth in the United States at 27 years, the use of DBS in pregnancy becomes an increasingly important topic (CDC, 2022). Additionally, many of the expanding indications for neuromodulation, such as major depressive disorder (MDD) and epilepsy, affect pregnant individuals. Each year, up to 20% of pregnant individuals suffer from a depressive disorder and over 1.1 million women of childbearing age have epilepsy (Sazgar, 2019; Van Niel and Payne, 2020). Uncontrolled and poorly controlled MDD and epilepsy in pregnancy pose significant threats to both mother and fetus, ranging from neurodevelopmental derangements to fetal hypoxia and growth restriction and even death (Battino and Tomson, 2007; Chan et al., 2014). In refractory cases of epilepsy (affecting 40% of all persons with the disease), seizures can increase in frequency during pregnancy (Kobau et al., 2008; Vitturi et al., 2019). Many first-line treatment modalities for these disorders, such as anti-epileptic pharmacotherapeutics, can cross the placenta and have known teratogenic effects on fetuses or neurodevelopmental delay during childhood, limiting the availability of safe and effective treatments in pregnancy (Artama et al., 2005). In these instances, the patient may benefit from alternative non-pharmacologic therapeutic approaches such as DBS.

It is thus an imperative to assess and analyze the data available surrounding DBS and the pregnant population with the most up to date evidence. Doing so will shed light on crucial areas of promise and progress, as well as better characterize considerations for each neurologist and obstetrician’s own practice. In this comprehensive review we will address pregnancy-related DBS concerns including clinical trial enrollment using all available studies in the published literature. Our primary objective was to describe clinical outcomes of pregnancies in which DBS was used. Our secondary objective was to assess how clinical trials approached participation eligibility during pregnancy, lactation, and the reproductive years.

## Methods

**FIGURE 1:**
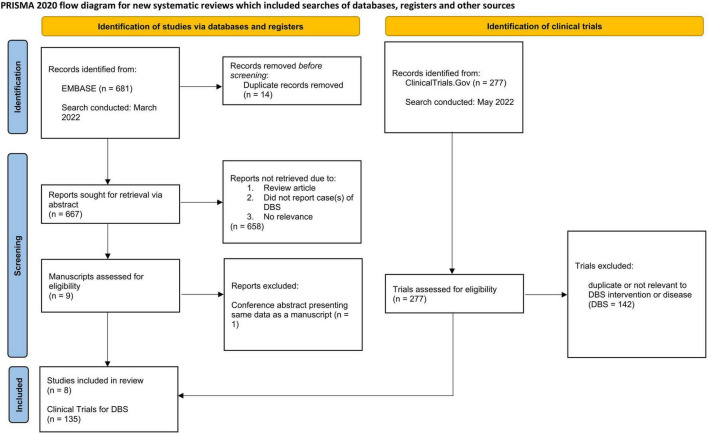
PRISMA 2020 flow diagram for systematic reviews detailing collection of data from EMBASE **(left)** and clinicaltrials.gov **(right)**.

A comprehensive review of the literature for DBS in the childbearing and pregnant populations was conducted from March 2022 to June 2022 querying EMBASE for all cases reported to date. Our search criteria is available in Supplementary Data Sheet 1. Two independent raters (CK and MP) conducted a preliminary survey of the literature search results, evaluating both title and abstract for initial relevance. The two raters then reviewed the manuscripts of the screened publications for rigor prior to inclusion in the analysis. Inclusion criterion for manuscripts were: (1) an original research article, case report, case series, or trial of DBS co-occurring with pregnancy, (2) reporting on a DBS case in pregnancy that was carried out to completion (i.e., the subject had given birth). Exclusion criteria were: (1) duplicate publications, or conference proceeding of an eventual manuscript, (2) review articles or non-research articles or (3) did not include pregnancy information within the article. Published abstracts, letters to the editor or publications from conference proceedings were included in accordance with the recommendations put forth by Scherer and Saldanha (2019) if they met relevant pre-determined criteria. The inclusion and exclusion criteria used for manuscripts was applied to abstracts and letters to the editor. The following data were extracted from each article: the condition to treat, DBS target structure, maternal complications, route of delivery, birth complications, neonatal complications, neurodevelopmental follow-up, and other pertinent information offered. This review was made in accordance with PRISMA guidelines as presented in Figure 1 (Page et al., 2021).

A search of ClinicalTrials.gov for DBS studies involving dystonia, pain, epilepsy, MDD, obsessive-compulsive disorder (OCD), and Tourette syndrome (TS) was also conducted. The same two independent raters reviewed the results and included trials meeting the following criteria:(1) involved DBS of the relevant clinical condition and (2) offered DBS-based intervention. A trial was excluded if it (1) was a duplicate of another trial; (2) focused on another condition outside of the search; (3) did not involve DBS; or (4) was observational and not pertaining to a recently offered DBS based intervention. For all included trials, each rater reviewed the trial’s inclusion and exclusion criteria. Three questions were addressed for each included trial. (1) Does the trial exclude subjects who are pregnant? (2) Does the trial exclude persons of childbearing age not on contraception deemed adequate by investigators and/or individuals intending to become pregnant? (3) Does the trial exclude breastfeeding individuals? Other data extracted from the clinical trial results included: enrollment status, regional location of trial, trial phase, date of trial start, and date of trial completion (if applicable).

## Results

### Deep brain stimulation

**FIGURE 2:**
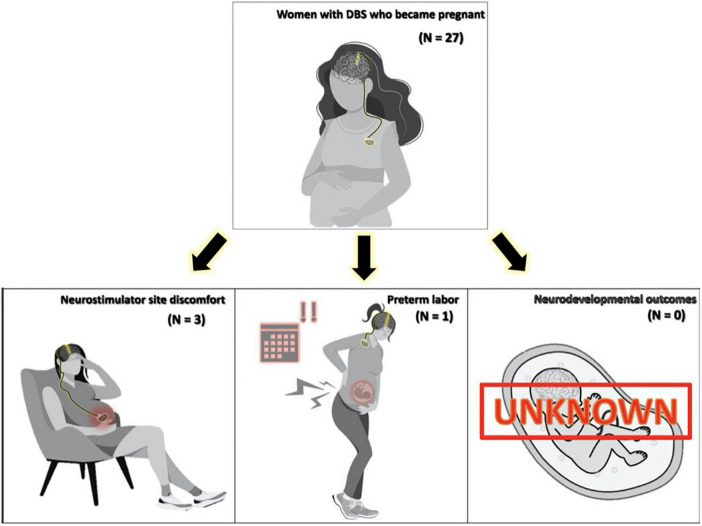
Figure displaying side effect profile found in a comprehensive literature review of pregnancy and Deep brain stimulation (DBS).

Results of the literature search are shown in Table 1 and illustrated in Figure 2. Out of 681 publications, there were eight eligible publications reporting DBS in pregnancy that were included for analysis. All eight were full-length original research articles. These eight publications reported information on 27 patients with DBS who subsequently became pregnant and all received active neuromodulation treatment throughout their pregnancy (Table 1). Two individuals became pregnant twice with DBS for a total of 29 pregnancies. Another two persons conceived twin pregnancies, which brings the total to 31 infants. The most prevalent condition was dystonia (*N* = 17, 63%) followed by epilepsy (*N* = 4, 15%) and PD (*N* = 3, 11%). The most frequent target was the globus pallidus internus (*N* = 20, 74%) followed by the anterior nucleus of the thalamus (*N* = 4, 15%) and the subthalamic nucleus (*N* = 3, 11%).

**TABLE 1:** Deep brain stimulation (DBS) literature review data.

Study (Author)	Subjects (*N*)	Infants (N)	Conditions* (N, %)	Target structure (N, %)	Pregnancy complications (N)	Follow-up (months)
Scelzo et al., 2015	11^a,b^	13	PD (3), TS (2), OCD (1), dystonia (5)	Bilateral Gpi (8), STN (3)	Stimulator site discomfort (2)	24
Bóné et al., 2021	2	2	Epilepsy	ANT		40.5
Ziman et al., 2016	6^a^	7	Dystonia	Bilateral Gpi	PTD at 35 weeks (1) + stimulator site discomfort (1)	50.8
Ozturk and Kadiroğulları, 2022	1	1	Dystonia	Bilateral Gpi		N/a
Paluzzi et al., 2006	3^b^	4	Dystonia	Bilateral Gpi		N/a
Park et al., 2017	1	1	Dystonia	Bilateral Gpi		36
House et al., 2021	2	2	Epilepsy	ANT		15
Lefaucheur et al., 2015	1	1	Dystonia	Bilateral Gpi		N/a
Total	27	31^c^	PD (3, 11%), TS (2, 7%), OCD (1, 4%), dystonia (17, 63%), epilepsy (4, 15%)	Bilateral GPi (20, 74%), STN (3, 11%), ANT (4, 15%)		Average (months) = 33.26

The table displays the results of the literature review for DBS in pregnancy, outlining the number of subjects/infants, conditions, target structures, and pregnancy complications for each of the included studies.

*PD, Parkinson’s disease; TS, Tourette’s syndrome; OCD, obsessive-compulsive disorder; GPi, globus pallidus internus; STN, subthalamic nucleus; SAB, spontaneous abortion; ANT, anterior nucleus of thalamus; PTD, preterm delivery, ^a^*n* = 1 subject became pregnant twice in this cohort, ^b^*n* = 1 subject had a twin pregnancy in this cohort, ^c^*n* = 1/31 fetus was spontaneously aborted in the first trimester determined to be unrelated to DBS.

Pregnancy complications for the 29 pregnancies were observed in three (12.9%) patients. Non-obstetric complications occurred in three individuals who had discomfort at the neurostimulator site (*N* = 2 subclavicular, *N* = 1 abdominal). One of these patients also had an obstetric complication of a preterm delivery at 35 weeks gestational age. The discomfort experienced by three subjects was mechanically induced from physiologic abdominal and breast changes as pregnancy progressed. One additional patient was reported to have experienced the spontaneous abortion of one fetus in the early weeks of a twin pregnancy (Scelzo et al., 2015). The authors, however, determined that this adverse event was not related to DBS, thus it was not included in our complication data. As noted by Ziman et al. (2016) a subject who experienced abdominal discomfort from the neurostimulator site was the same individual who experienced preterm birth at 35 weeks. This patient had a battery readjustment procedure following her first pregnancy, relocating the battery from the abdomen to the subcutaneous tissue of the chest. With her subsequent second pregnancy, she experienced less discomfort and had a term delivery. Three publications did not include any follow-up data on neonatal outcomes. The remaining publications (*N* = 5) followed subjects from 6 to 108 months. No negative neurodevelopmental outcomes were reported.

No articles were found focusing specifically on DBS’s effects on lactation or future fertility; however all subjects had a DBS stimulator prior to conception and reported no issues with becoming pregnant. Additionally, five out of eight publications followed DBS subjects from initiation of DBS treatment, into a pregnancy, and then into the postpartum period. These studies did not report any issues related to fertility. One subject reported inability to breastfeed due to discomfort. Otherwise, there were no reports in the literature of DBS directly affecting lactation.

Out of a potential of 277 clinical trials for the secondary objective, 135 trials on DBS met criteria (Figure 1). In clinical trials offering DBS lead implantation across all conditions, 68% (*N* = 92) excluded actively pregnant subjects, 22% (*N* = 29) excluded breastfeeding individuals, and 44% (*N* = 59) excluded persons of childbearing age intending to conceive within the coming years and/or individuals not on “adequate” contraception. Details on which contraceptive methods were deemed adequate were not specified in the exclusion criteria. Trials examining DBS for MDD were most likely to exclude the populations of interest (91%). The trial indication least likely to exclude this population was dystonia (41%) (Table 2).

**TABLE 2:** Clinical trials review data.

	Condition	Included trials	Excludes pregnancy	Excludes POCBA*	Excludes breastfeeding
DBS					
	Pain	10	8 (80%)	6 (60%)	4 (40%)
	Dystonia	34	14 (41%)	4 (21%)	7 (21%)
	Tourette’s	11	7 (64%)	3 (27%)	4 (36%)
	Epilepsy	15	11 (73%)	10 (67%)	2 (20%)
	Depression	35	32 (91%)	25 (71%)	6 (17%)
	Total (DBS)	**135**	**92 (68%)**	**59 (44%)**	**29 (22%)**

The table details the exclusion of various populations from clinical trials by modality of neuromodulation and the condition of interest for each trial.

*Persons of childbearing age (POCBA) planning to conceive or not on “adequate” contraception.

## Discussion

Though our literature search for our primary objective to describe patients with DBS and pregnancy returned 681 potential articles, only eight met inclusion criteria for analysis. Data from these 8 reports of 29 pregnancies and 31 infants suggested reasonable safety profile for DBS in pregnancy. The 1 preterm birth (1 of 29 pregnancies, 4%) seems unlikely to be related to the DBS intervention since the expected rate of preterm birth in the general population would be approximately 10–12% (Walani, 2020). Discomfort at the battery site occurred in three pregnancies (10%) and may be a risk given the physiologic body changes related to pregnancy. Aside from the abdominal site, the nature of discomfort experienced by the patients with pulse generators in the subclavicular region is curious. This may suggest migration of the DBS pulse generator in response to pregnancy related body habitus changes. BMI was not discussed in the studies reporting neurostimulator site discomfort. All 27 patients had DBS leads implanted prior to pregnancy with no reported fertility issues across a variety of subcortical targets.

The explicit exclusion of pregnant individuals from 68% of interventional trials involving DBS is somewhat problematic. It is a legitimate concern to proceed with caution when there are questions whether there is adequate equipoise for including this vulnerable population given the paucity of data. Based on our review, there were no trials, including observational, specifically designed to study the safety or efficacy of DBS in the pregnant population. This impairs the ability to determine whether DBS could be a reasonable and effective intervention in pregnancy. Nevertheless, our data are reassuring that there are no clear significant safety signals as of yet. Importantly, there are data from other treatments demonstrating relative safety and effectiveness of interventions that use electrical stimulation, such as cardioversion for arrhythmias and electroconvulsive therapy (ECT) in pregnancy (Yonkers et al., 2009; Enriquez et al., 2014). Interestingly, when reviewing the clinical trials for DBS in MDD, very specific inclusion criteria were utilized such that the pregnant and post-partum populations, of which there is a significant incidence and prevalence, were excluded One possible explanation for this could be the attempt to limit confounding variables given the historically variable response after DBS in the already very specific treatment resistant depression population.

**FIGURE 3:**
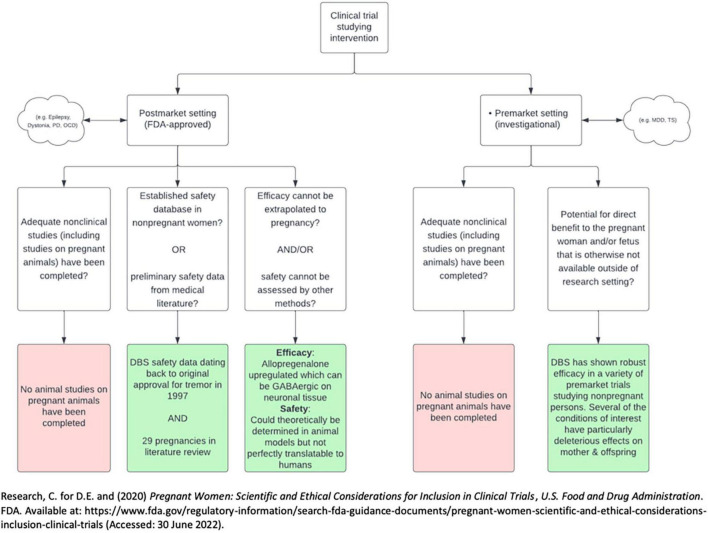
Flow diagram describing 2018 draft by FDA on the inclusion of pregnant persons in clinical trials. PD, Parkinson’s disease; OCD, obsessive-compulsive disorder; MDD, major depressive disorder; TS, Tourette’s syndrome.

It is unlikely that experience with DBS in only 29 pregnancies is sufficient to offer guidance on DBS and pregnancy. For pharmacotherapeutics, the Food and Drug Administration (FDA) typically requires data from clinical trials adequately powered before offering clinical guidance. Because DBS trials are designed to treat a specific indication, the only way to learn whether DBS should be a treatment option in pregnancy is to include (not exclude) pregnancy in treatment trials for those indications. This is consistent with the FDA’s stance on research and pregnancy. In 2018 the FDA issued a draft of a guidance communicating there were high exclusion rates of pregnant persons in clinical trials (Center for Drug Evaluation and Research, 2020). Guidance documents can be used to communicate regulatory hurdles and suggestions for industry to navigate the hurdles. This draft was revised in 2019, and has since seen no activity in revisions or suggestions. Specific medical devices were not mentioned in this document, which focused on pharmacologic agents. In the guidance, a framework was proposed by the FDA for inclusion of pregnant individuals in trials studying pharmacologic agents, which perhaps could be modeled to guide medical device trials in pregnancy. The FDA suggested the inclusion of pregnant individuals in clinical trials is ethically justifiable under specific criteria. These criteria, which differ slightly if the clinical trial is classified as pre- or post-market, are outlined in Figure 3.

There are several important issues to consider regarding potential for adverse effects of DBS in pregnancy. There are no animal models studying the safety of DBS in pregnancy and lactation or the effects of DBS on fertility. The first criterion for both pre-market and post-market studies from the FDA’s draft for the inclusion of pregnant individuals in clinical trials thus has not been met. Furthermore, the mechanism of action of DBS is not fully delineated, making it difficult to anticipate potential downstream effects on the reproductive cycle (Herrington et al., 2016). There is a paucity of evidence that DBS may affect second messengers involved with reproductive physiology. For example, DBS of the nucleus accumbens (not a target structure in any study in our review) has been shown to result in shifts in prolactin and cortisol levels (de Koning et al., 2013, 2016). Another DBS target with potential reproductive consequences is the hypothalamus. DBS targeting the lateral hypothalamic nuclei has been used to treat obesity, cluster headache, generalized anxiety disorder, and post-traumatic stress-disorder (May, 2008; Franco et al., 2018; Li et al., 2022). There is potential for off-target stimulation of other hypothalamic nuclei. These off target effects could theoretically impact fertility, pregnancy, and lactation. Finally, pregnancy induces the production of a large amount of allopregnanolone, a steroid hormone with gamma aminobutyric acid (GABA)-ergic effects on the central nervous system. Given higher levels of GABA-like activity during pregnancy, pregnant individuals may theoretically require pregnancy-specific stimulation settings to successfully achieve a therapeutic effect (Kim et al., 2011).

When considering these issues, it may be helpful to examine the status of ECT in pregnancy as it is relevant to the discussion about DBS. ECT has been approved for use in pregnancy and there is little ongoing debate regarding its safety in this population. No animal model studies were provided to support the use of ECT in pregnancy. This safety determination was made jointly by amassing case reports in 1994, and the American College of Obstetricians and Gynecologists (ACOG) and the American Psychiatric Association confirmed the evidence in 2009 (Miller, 1994; Yonkers et al., 2009). A review by Rose et al found four meta-analyses studying ECT, three of which found similar numbers of cases in the literature of over 300 (Rose et al., 2020). The most recent (fourth) meta-analysis in 2014 removed cases before 1975, which marked the transition to modern ECT anesthesia away from the insulin coma. This eliminated confounding adverse effects, and yielded only 76 cases (Pompili et al., 2014). This process could serve as a framework for determining the safety of neuromodulation in pregnant or lactating individuals. We encourage obstetricians, neurologists, psychiatrists, and neurosurgeons to continue publishing more cases of DBS in pregnancy to strengthen the body of evidence.

The results of this comprehensive review should be interpreted with caution. Included studies were retrospective case reports or case series. Heterogeneity of cases, sample size, limited follow up, outcome reporting bias, and publication bias could all have impacted the results. Additionally, the reported neonatal and infant outcome metrics were inconsistently reported. The data collected on child neurodevelopment was heterogenous, with some data collected up to 108 months and other studies failing to report any follow-up. Follow-up data, when available, varied from gross assessments to more specific milestone assessments conducted specifically by pediatricians. Further studies would be strengthened by uniform and detailed protocol assessments of neurodevelopmental progress. Nevertheless, the risk to the individual or fetus remains theoretical as the literature does not demonstrate evidence of harm beyond average obstetric risk. It is not theoretically feasible for electrical current from DBS stimulation to reach the uterus or developing fetus. There is little evidence to support the hypothetical risk of target stimulation leading to direct downstream effects on pregnancy, lactation, or fertility, similar to the use of ECT. The current clinical data for DBS in pregnancy are largely reassuring, demonstrate no clear adverse safety signals, and the sample size is large enough to justify inclusion of pregnant patients in well-designed clinical trials. Performing such trials is critically important to facilitate understanding of whether DBS has a role as an intervention in pregnancy and in reproductive age-patients. Without such studies, pregnant patients could be denied potentially effective treatment for serious conditions, which themselves could adversely impact obstetric and pediatric outcomes. Taking the complication rate of 12.9% in our cohort treated with DBS becomes particularly poignant when compared to, for example, a cohort of pregnant patients with dystonia without DBS experienced complications during pregnancy or delivery at a 45.26% rate (San Luciano et al., 2019). It is thus an imperative to find an inclusive set of criterion that allow for the access of a the latest treatment modalities to those most vulnerable.

## Conclusion

The data from 29 pregnancies in 27 subjects suggest that DBS during pregnancy does not have a high perinatal complication profile. The most common reported concern was device discomfort, which should be considered when planning device placement in individuals considering pregnancy. Many but not all clinical trials exclude pregnant individuals and the documentation of safety in larger numbers of subjects may make more clinical trials available for pregnant individuals in the future. Increasing the number of pregnancy-related publications will clarify the safety profile for individuals with DBS interested in becoming pregnant and those who may find out they are pregnant following DBS implantation. Though the safety profile is emerging, the still small number of cases has hampered regulatory agencies from offering clear guidance on safety and on inclusion of this population in clinical trials. It will be interesting to observe whether the guidance will be similar to that for ECT. A roadmap guiding investigators toward safe neuromodulation in pregnant and lactating individuals will be of utmost importance, as DBS continues to expand indications, and other neuromodulation techniques gain popularity (e.g., transcranial magnetic stimulation and transcranial direct current stimulation).

## Data availability statement

The raw data supporting the conclusion of this article will be made available by the authors, without undue reservation.

## Author contributions

CK and TP contributed to drafting the manuscript and data extraction. CK, TP, KR-R, AR-Z, JS, MO, and JW contributed to analysis and interpretation of the data in their respective expertise. AR-Z, KR-R, JS, MO, and JW critically revised the manuscript. All authors approved the manuscript as submitted.
